# N-Acetyl Cysteine as a promising therapeutic approach in ovarian cancer: potential and perspectives

**DOI:** 10.20935/acadonco7784

**Published:** 2025-06-24

**Authors:** Erin A. Kindlon, Graham P. Pidgeon

**Affiliations:** 1 Translational Oncology, Trinity Translational Medicine Institute, Trinity College Dublin, Dublin D02PN40, Ireland.; 2 Department of Surgery, Trinity Cancer Institute, St James’s Hospital & Trinity College Dublin, Dublin D08W9RT, Ireland.

**Keywords:** N-Acetyl Cysteine, ovarian cancer, chemotherapy, toxicity, clinical trials

## Abstract

Ovarian cancer is the seventh most common cancer in women and the eighth most common cause of cancer death worldwide. It is an aggressive disease with five-year survival rates below 45% and many patients relapse within 2 years. Further treatments become more intense, resulting in chemotherapy drug resistance and increased toxicity. This has created the need to develop new therapeutic strategies to improve the quality of life and treatment options for ovarian cancer patients. Studies have reported the role of cysteine in ovarian cancer, primarily as a precursor of glutathione (GSH), contributing to the endogenous antioxidant mechanism. The membrane-permeable cysteine precursor N-acetylcysteine (NAC) can enhance the intracellular cysteine pool and thus results in decreased oxidative stress. This characteristic provides NAC with a rationale as a potentially effective chemo-protectant in ovarian cancer treatment. In this review, we summarize the effects of NAC supplementation in ovarian cancer from recent preclinical studies. The role of NAC in chemotherapy response, and mechanisms to overcome chemo resistance in ovarian cancer (including targeting the Mirk/dyrk1B kinase pathway) are also explored. While NAC holds therapeutic promise in alleviating treatment-associated toxicities, its application in ovarian cancer requires careful consideration based on tumour subtype, redox context, and treatment timing. Future research incorporating subtype-specific models and clinical trials will be essential to delineate the precise role of NAC and optimize its integration into ovarian cancer treatment regimens.

## 1. Introduction

Chemotherapy drugs have improved the survival rates of cancer patients and play a significant role in the treatment of most cases. However, given that chemotherapy is cytotoxic, patients often suffer side effects during and after treatment [1]. Despite ongoing advances in the development of targeted therapies with reduced toxicity to normal tissues, chemotherapy continues to serve as a vital component of cancer treatment and is likely to remain so for the near future [2]. This reality highlights the imperative to optimize quality of life for patients undergoing chemotherapy and to implement strategies aimed at preventing the onset of significant treatment-related impairments.

A new area of research which has gained attention in recent years is the role of antioxidants in the setting of cancer. Human antioxidant defence functions to maintain an ideal cellular redox homeostasis by maintaining reactive oxygen species (ROS) at a minimal level to still allow ROS to conduct necessary cell signalling [3]. Most research in this area has focused on the presence of antioxidant foods in the diet and their potential in preventing cancer development [4]. While ROS are an unavoidable byproduct of conserved cellular processes, an excessive amount of ROS can lead to oxidative stress which results in both toxic effects and DNA damage. Recent studies have started to investigate the effect of antioxidant supplementation in cancer treatment with N-acetylcysteine (NAC), the L-cysteine precursor, used most frequently in these studies [5] (Table 1).

Currently, the use of antioxidants including NAC in cancer treatment is controversial. While reducing ROS levels can prevent DNA damage and toxicities, it can also reduce treatment efficacy. A fundamental mechanism of chemotherapeutics is that they induce ROS-mediated cell injury in cancer, leading to cell death [6]. Thus, inhibiting ROS levels may facilitate cancer cells to evade cell death.

NAC has demonstrated paradoxical effects in various cancer models. In mouse melanoma models, pretreatment of NAC increased tumour formation tenfold while also protecting against the deleterious effects of oxidants in metastatic cells, suggesting that NAC is a contraindication for melanoma [5]. In glioblastoma models, NAC prevented proliferation, invasion, and migration in an antioxidant-independent manner by targeting Notch2 [7].

This report explores the role of cysteine in the context of chemotherapy for ovarian cancer, examining the potential of N-acetylcysteine supplementations to mitigate chemotherapy-induced toxicities and potentially enhance treatment outcomes. Strategies aimed at inhibiting cysteine metabolism that are currently being evaluated for their capacity to overcome chemoresistance and improve therapeutic efficacy will be detailed.

## 2. N-acetyl cysteine in ovarian cancer

NAC is a synthetic product of the amino acid, L-cysteine, commonly prescribed to detoxify acetaminophen overdose due to its antioxidant properties [8]. During detoxification, it assists in the replenishment of the Glutathione (GSH) store in hepatocytes. Cysteine is the rate-limiting step in GSH synthesis; therefore, NAC supplementation increases the intracellular cysteine pool and increases GSH detoxification activity of H_2_O_2_ [5]. NAC has been shown to increase the serum levels of GSH in several disease linked to oxidative stress due to GSH depletion [9]. Furthermore, NAC also has direct antioxidant activity through its free thiol group, which interacts with reactive oxygen species (ROS) [10]. Other antioxidant properties of NAC include its ability to help regulate the redox state by breaking disulfide bonds to restore thiol stores, reduce inflammation markers and oxidative stress, and inhibit mitochondrial transfer by ROS scavenging [11]. Chemotherapy increases both primary toxicants and reactive electrophiles that arise as metabolites or lipid peroxidation products which are inactivated with NAC supplementation, indicating its chemoprotective potential [11].

So far, the studies concerning NAC as a treatment strategy in ovarian cancer patients have led to the hypothesis that antioxidant supplementation during chemotherapy should be effective in reducing chemotherapy-induced toxicities. However, limitations of this approach have also been reported, suggesting that minimizing the side effects of cytotoxic drugs also reduces their effectiveness on cancer cells, compromising overall treatment efficacy [12].

The clinical use of platinum-based chemotherapeutic agents is limited in ovarian cancer due to their severe toxicity and dose-dependent side effects. Agents such as cisplatin and carboplatin are a cornerstone of the treatment given to patients with ovarian cancer, particularly at high dose, for curative intent [5]. The side effects of these drugs are frequent and diverse, including cognitive impairments, nephrotoxicity, neurotoxicity, and ototoxicity [11]. In other cancer types, such as non-small cell lung cancer, NAC has been shown to protect against neurotoxicity caused by anti-cancer agents. NAC inhibited the expression of nuclear transcription factor kappa-B (NF-kB) and reduced neurological damage caused by disulphiram (DSF) [13].

Table 1 shows both in vivo and in vitro studies of NAC in ovarian cancer models to date. NAC has been shown to have a chemoprotective effect in multiple studies, without affecting the anti tumour efficacy of cisplatin and doxorubicin. Other studies have shown that NAC rescuing of cysteine stores reduces the rate of cytotoxic-induced cancer cell death. Similar effects were seen in studies of NAC in combination with radiation, which preserved ovarian tissue.

## 3. N-acetyl cysteine potentiates chemotherapy

Cisplatin was found to decrease the levels of GSH in both the frontal cortex and hippocampal of ovarian cancer rat models [5]. The depleted GSH levels are associated with increased oxidative stress, resulting in cognitive decline. In this study, the delayed administration of NAC sustained the cross-linking activity of cisplatin in rapidly dividing cells while protecting non-cancerous neural cells which are less rapidly dividing. NAC utilizes several protective mechanisms against platinum chemotherapeutics. It counteracts the depletion of GSH by acting as a source of L-cysteine for increased GSH synthesis. It can also indirectly increase free cysteine available for GSH synthesis by regulating plasma and tissue protein-bound cysteine levels through thiol exchange [21]. As a result, NAC prevented cognitive dysfunction without reducing survival or promoting tumour growth in ovarian cancer rats [5]. This study is the first ovarian rat model of CRCI prevention by NAC, which will be further investigated in the first human clinical trial NCT04520139. This phase, 1/2, which is estimated to begin in December 2025, aims to determine the maximum tolerated dose and safety and tolerability of adding NAC to ovarian cancer patients who are receiving a platinum-based therapy. It will consist of 2 arms (1) platinum-based therapy + NAC (2) platinum-based therapy + placebo, to investigate whether NAC will mitigate chemotherapy-related cognitive impairment.

NAC has also been shown to scavenge reactive oxidants through direct interaction via its thiol group. While this mechanism appears to play a role in reducing cytotoxicity in ovarian cancer cells, it may concurrently inactivate intracellular cisplatin by interfering with the platinum–DNA binding process [22]. This raises an important question: does the protective effect of NAC come at the cost of diminished chemotherapeutic efficacy in ovarian cancer cells?

**Figure 1 •:**
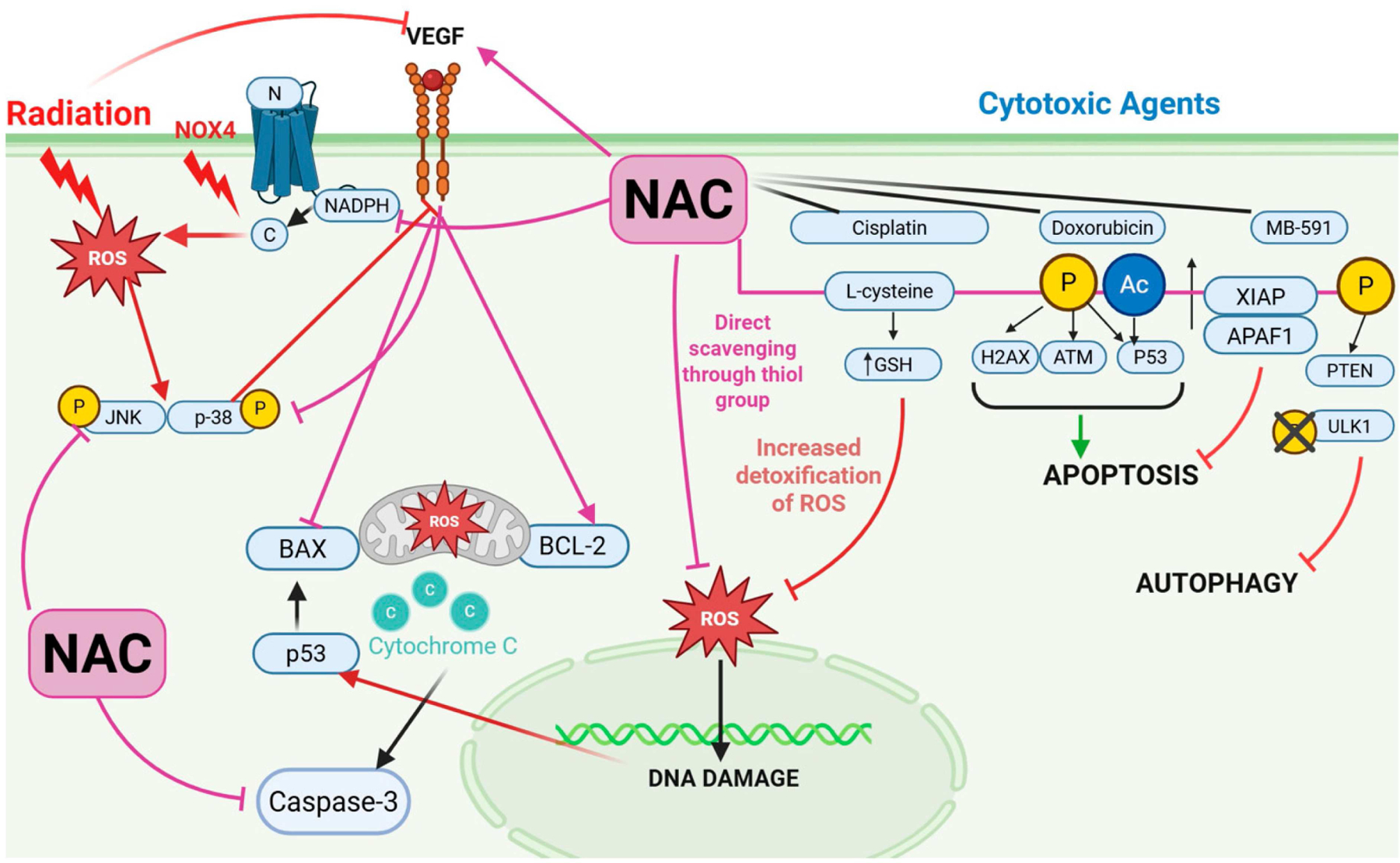
The effects of N-Acetyl Cysteine (NAC) supplementation on the cellular response to cytotoxic agents and radiation. NAC alleviates radiation-induced oxidative damage through inhibition of NADPH subunits and preserves ovarian function through up-regulation of VEGF expression and suppression of NOX4/MAPK/p53 signalling. NAC exhibits anti-apoptotic effects against radiation by decreasing cytochrome c and caspase 3 levels. NAC exhibits direct antioxidant activity against ROS by scavenging through its thiol group. Chemoprotective abilities of NAC include increased detoxification of ROS through GSH synthesis, potentiates doxorubicin-mediated apoptosis lowering the dosage needed, inhibition of apoptosis and autophagy when combined with MB-591. Created in https://BioRender.com.

There is a paradoxical effect observed in cisplatin-sensitive (A2780) vs. cisplatin-resistant ovarian cancer cells (A2780 cis) when NAC is administered [23], with NAC having an anti-apoptotic effect, particularly in A2780 cells, by exclusively expressing the apoptotic inhibitor protein XIAP [14] while in A2780 cis cells, NAC primarily inhibits the PTEN/Akt/mTOR pathway through phosphorylation of PTEN and ULK1 to inhibit the autophagy initiation [14] (Figure 1). Previous studies of NAC in ovarian cancer cells have regarded this cancer as a single entity, without treating each subtype separately. Understanding that NAC has different effects depending on the treatment sensitivity, suggests that the same could be true in different ovarian cancer subtypes as they respond differently to chemotherapy [24]. More clarity could be gained on the role of NAC in ovarian cancer by treating each subtype as a separate disease.

Another chemotherapy drug used to treat ovarian cancer is the potent antineoplastic agent, Doxorubicin (Dox) [25]. Dox induced cell death in cancer cells by causing both DNA damage and activating various regulatory mechanisms inducing apoptosis or mitotoic catastrophe [26, 27]. The ATM/p53 pathway has been shown to be activated by Dox, resulting in ROS-mediated cell death [15]. When ovarian cancer cells were treated with NAC prior to Dox treatment, it enhanced ATM and p53 phosphorylation, potentiating the effect of Dox on ovarian cancer cells [28]. Furthermore, NAC has demonstrated a protective ability against doxorubicin-induced toxicities such as cardiotoxcity and hepatoxicity [29–31].

## 4. Limitations of N-acetyl cysteine and chemotherapy

Contradictory studies have reported that NAC can have a diminishing effect on chemotherapy efficacy, which may limit its use in ovarian cancer. NAC reduced the efficacy of Dox treatment by two mechanisms [32]. (1) NAC enhanced MRP1-mediated Dox resistance through GSH synthesis in both cancer and normal cells [33]. (2) The antioxidant properties of NAC resulted in reduced ROS levels which are needed for ATM activation upon doxorubicin treatment [31]. The combination of NAC and Dox in ovarian cancer is yet to be evaluated in human trials. Dresdale et al. assessed this combination in sarcoma patients and found that N-acetal cysteine had no effect in reversing doxorubicin-induced cardiomyopathy [34].

Cysteine was found to protect ovarian cancer cells from the adverse hypoxic microenvironment and platinum-based chemotherapy, thus contributing to cancer progression [18]. In particular, after two cycles of carboplatin, cysteine was found to protect ovarian cancer cells from carboplatin-induced death in both hypoxic and normoxic conditions. Carboplatin only induced higher cell kill in the absence of cysteine treatment, suggesting that cysteine allows for faster adaption to carboplatin and may limit its efficacy.

## 5. Cysteine-mediated treatment resistance in epithelial ovarian cancer subtypes

Epithelial ovarian cancer (EOC) encompasses a heterogenous group of histological subtypes, including serous (OSC), clear cell (OCCC), endometrioid, and mucinous carcinomas, each with distinct molecular characteristics and variable responses to chemotherapy [35]. Disease stage and subtype significantly influence treatment outcomes, particularly in the context of platinum-based chemotherapy.

**Figure 2 •:**
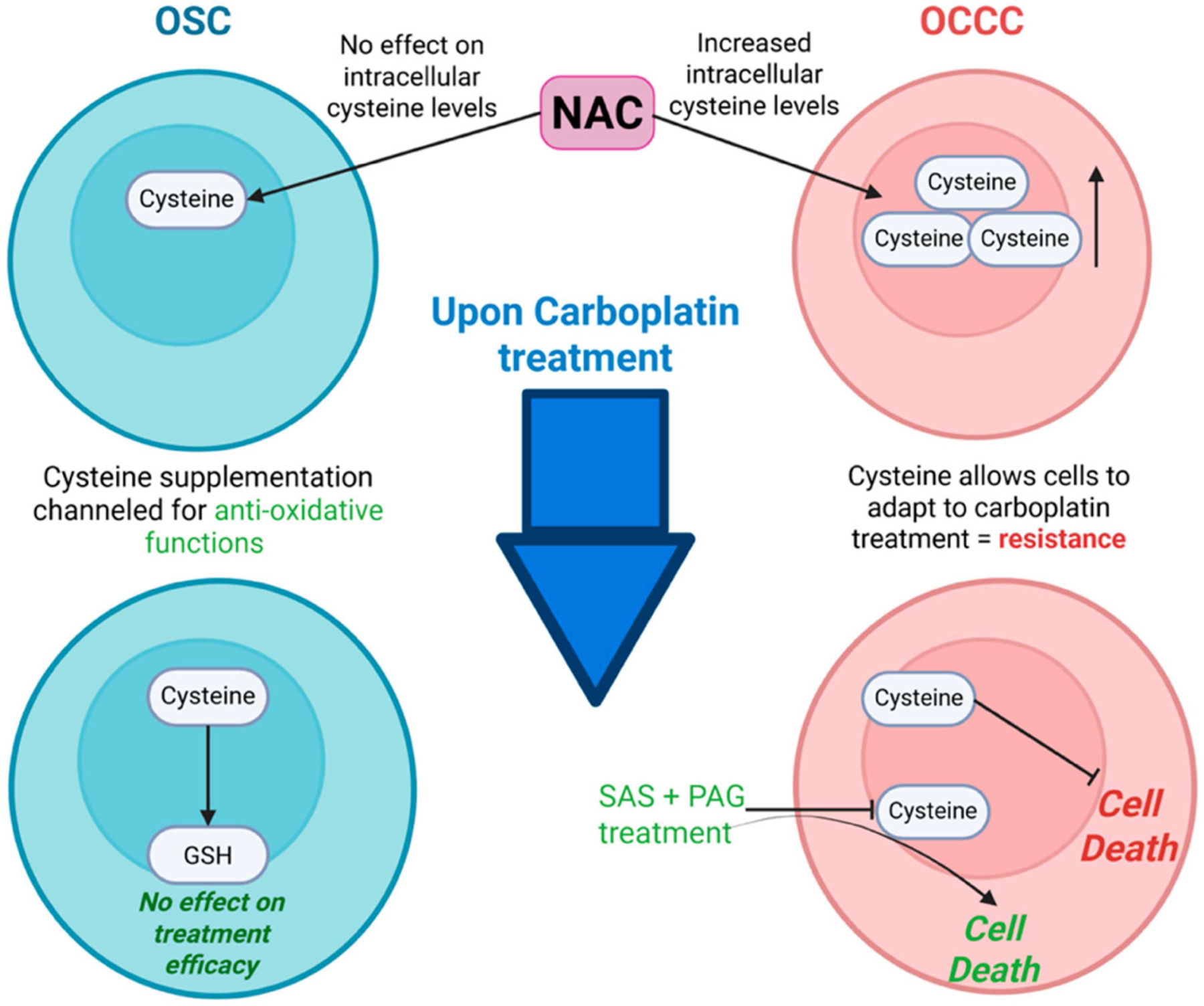
Different activity of NAC between ovarian serous carcinoma (OSC) vs. ovarian clear cell carcinoma (OCCC) cell lines. NAC supplementation in OCCC cell lines increases intracellular levels of cysteine facilitating cell survival and treatment resistance. Cysteine depletion approaches (SAS+ PAG treatment) reverse these effects and allows for carboplatin-induced cell death. NAC treatment in OSC cell lines has no effect on the intracellular cysteine levels. The supplemented cysteine is channelled for anti-oxidative functions with no effect on treatment efficacy. Created in https://BioRender.com.

Emerging evidence indicates that elevated levels of cysteine and cysteine-rich metabolites, such as glutathione (GSH), are associated with resistance to platinum compounds, including carboplatin and cisplatin [32, 36]. In highly chemo-resistant OCCC lines, supplementation with exogenous cysteine has been shown to significantly elevate intracellular free cysteine levels, thereby conferring protection against carboplatin-induced cytotoxicity under both hypoxic and normoxic conditions [37, 38]. These findings suggest that cysteine not only mitigates drug-induced cell death but also facilitates metabolic adaptation to carboplatin, enabling tumour cell survival despite therapeutic pressure. Notably, increased cell death was only observed upon the withdrawal of cysteine supplementation, underscoring its critical role in promoting chemoresistance (Figure 2).

In contrast, (OSC) cell lines had lower baseline levels of intracellular cysteine, and exogenous cysteine supplementation did not alter intracellular cysteine pools. Instead, the supplemented cysteine is primarily utilized for the biosynthesis of antioxidant molecules such as GSH, without significantly altering sensitivity to carboplatin [18]. These subtype-specific differences in cysteine metabolism highlight the divergent cellular handling and functional consequences of cysteine supplementation in ovarian cancer, suggesting that it may not be appropriate for OCCC due to its potential to reduce chemotherapy effectiveness.

The rare histotype ovarian clear cell carcinoma (OCCC) is known to be resistant to conventional platinum-based therapy. It is molecularly defined by hepatocyte nuclear factor 1 beta (HNF1B) overexpression and PIK3CA mutations [39]. However, these markers do not have any predictive value for treatment outcomes in clinical scenarios. A recent study by Novera et al. revealed that OCCC has a profound reliance on cysteine for growth and may conduct other roles in addition to its anti-oxidative function [16]. In this subtype, intracellular cysteine accumulation acts as a resistance mechanism, which could be a potential target, rather than a favourable antioxidant strategy.

## 6. Mechanisms for overcoming chemoresistance in ovarian cancer

### 6.1. Targeting redox homeostasis and apoptosis in OCCC via cysteine depletion

Depleting cysteine stores with SAS and PAG combination treatment (SP) both in vitro and in OCCC mice models successfully inhibited tumour growth, with no side effects reported (Figure 2). A dual cytotoxic mechanistic action of cysteine deprivation was noted: (1) apoptosis independent of oxidative stress and (2) oxidative stress-dependent necrosis in glycolytic cells, resulting in necrosis and ferroptosis [16]. The pre-addition of NAC alone, but not other anti-oxidative agents, prevented the cytoxicity of the cysteine depletion, highlighting the role of cysteine availability for OCCC cell survival. Another recent strategy proposed to combat cysteine-mediated cisplatin resistance in OCCC is the depletion of other key players such as Mirk kinase to increase ROS levels in the cell [40].

### 6.2. Mirk kinase as a redox modulator and therapeutic target in ovarian cancer

Mirk/dyrk1B kinase is minimally expressed in most normal tissues but is frequently upregulated or genomically amplified in many ovarian cancers, suggesting a tumour-specific role in disease pathogenesis and therapeutic resistance [41]. Mirk has been shown to transcriptionally regulate a network of antioxidant genes, thereby maintaining low intracellular reactive oxygen species (ROS) levels in tumour cells. Consequently, inhibition or depletion of Mirk results in a marked increase in intracellular ROS, sensitizing cancer cells to oxidative stress-inducing therapies such as cisplatin. Experimental data across all four major ovarian cancer subtypes indicate that Mirk depletion enhances cisplatin responsiveness, even at low drug concentrations, suggesting that this approach may simultaneously improve therapeutic efficacy and reduce cisplatin-associated toxicities [17]. The authors reported that co-treatment with N-acetylcysteine (NAC) partially rescued cell viability in both Mirk-depleted and control cells treated with cisplatin, underscoring the critical role of redox modulation in Mirk-targeted strategies. However, this investigation was limited to in vitro cell line models, and the potential impact of this combination on systemic toxicities remains to be elucidated in preclinical or clinical settings.

Additional mechanistic insights reveal that Mirk has limited function in actively cycling cells and predominantly exerts its effects in quiescent cells. Specifically, Mirk phosphorylates components of the DREAM complex, a key regulator of G0/G1 cell cycle arrest, thereby promoting the survival of non-proliferating tumour cells [42]. This is of clinical interest, as quiescent tumour cells often evade the cytotoxic effects of conventional therapies, including radiation and chemotherapy. Targeting Mirk in this cellular compartment may therefore provide a strategy to eradicate treatment-refractory cell populations.

Further studies have demonstrated that Mirk depletion leads to increased expression of cyclin D1, facilitating cell cycle re-entry and promoting transition into the S phase, a stage more vulnerable to DNA-damaging agents [40]. This cell cycle modulation, in combination with enhanced ROS levels, contributes to the observed increase in cell death upon Mirk inhibition. However, the cytotoxic effects of Mirk depletion were again mitigated by concurrent NAC treatment, emphasizing the antagonistic interaction between ROS elevation and antioxidant supplementation.

Collectively, these findings support the therapeutic potential of targeting Mirk kinase to overcome chemoresistance in ovarian cancer, particularly by eliminating quiescent tumour cells. Nonetheless, the translational relevance of these findings requires further validation in more physiologically representative models, as current evidence is derived exclusively from in vitro systems. Future studies using patient-derived xenografts or organoid models are warranted to evaluate both the efficacy and safety of Mirk-targeted strategies in combination with redox-modulating agents such as NAC.

## 7. The role of N-acetyl cysteine in immunotherapy

Immunotherapy represents a promising therapeutic modality in the treatment of gynecological malignancies and may soon emerge as a superior alternative to conventional systemic chemotherapy [43]. Despite its potential, immunotherapeutic strategies in ovarian cancer remain in early clinical development, with currently observed response rates being suboptimal. Additionally, the use of immune-based therapies is often associated with immune-related adverse events (irAEs), necessitating the investigation of combination strategies to enhance efficacy and manage toxicity [44].

N-acetylcysteine (NAC), as mentioned previously is a well-characterized antioxidant, however it has also been shown to modulate immune responses, particularly through its regulatory effects on CD8+ T cells. In vitro studies indicate that NAC promotes CD8+ T cell differentiation and enhances their proliferative and cytotoxic functions, which may contribute to improved anti-tumour activity [43, 45]. Various studies have shown that NAC demonstrates immunomodulatory effects when combined with PD-1 blockers, chimeric antigen receptor (CAR)-T cell therapy and adoptive cell therapy.

In mouse colorectal cancer models, NAC demonstrated a synergistic effect with PD-1 blockers against tumour progression by promoting CD8+ T cell glucose metabolism and inducing TCF1+PD1+CD8+ T cell differentiation. Although PD-1 blockade is under active investigation in ovarian cancer, its clinical efficacy remains modest [46] (see Table 2). Future research should therefore explore the adjunctive use of NAC with PD-1 inhibitors as a means to optimize therapeutic outcomes in ovarian cancer. CAR-T cell therapy has demonstrated transformative success in hematologic malignancies; however, its efficacy in solid tumours such as ovarian cancer remains limited. Ongoing clinical trials have primarily targeted tumour-associated antigens including mesothelin and MUC16 (Table 3). A major barrier to CAR-T cell efficacy in solid tumours is the phenomenon of T cell exhaustion within the tumour microenvironment [47]. Preclinical studies indicate that NAC can reverse oxidative stress-induced T cell exhaustion, thereby enhancing CAR-T cell expansion and persistence [48]. In melanoma models, NAC supplementation was shown to restore effector function in exhausted T cells during adoptive T cell therapy, primarily through activation of the PI3K/Akt pathway and suppression of Foxo transcription factors [49]. These findings highlight the potential of NAC to augment the efficacy of adoptive immunotherapeutic approaches. Table 4 provides an overview of current clinical trials investigating adoptive immunotherapy in ovarian cancer.

## 8. Conclusions

Cysteine availability plays a critical role in modulating treatment outcomes in ovarian cancer, serving both as a metabolic vulnerability and a potential therapeutic avenue through supplementation with N-acetylcysteine (NAC). NAC, a cysteine prodrug, has demonstrated several beneficial properties, including the mitigation of chemotherapy- and radiotherapy-associated toxicities such as cancer-related cognitive impairment. Additionally, NAC has been shown to potentiate the cytotoxic efficacy of doxorubicin, enabling effective tumour cell killing at reduced drug concentrations. These findings suggest that NAC may enhance therapeutic windows by improving tolerability and efficacy in certain ovarian cancer contexts.

Despite these promising outcomes, the clinical utility of NAC remains complex and context-dependent, with divergent effects observed across ovarian cancer subtypes. In clear cell carcinoma of the ovary (OCCC), a subtype characterized by inherent resistance to platinum-based chemotherapy, increased antioxidant capacity through NAC supplementation may be detrimental. The elevation of intracellular glutathione (GSH) levels via NAC can further dampen the efficacy of oxidative stress-inducing chemotherapeutics, potentially exacerbating treatment resistance. Conversely, in ovarian serous carcinoma (OSC), NAC-derived cysteine appears to be efficiently utilized for GSH biosynthesis without significantly impairing chemotherapeutic activity, suggesting a more nuanced subtype-specific response to antioxidant therapy.

Notably, combinatorial strategies involving the dual depletion of mirk kinase and cysteine have shown efficacy in overcoming chemoresistance in ovarian cancer cell lines. This pro-oxidant therapeutic approach induces cancer cell death and is antagonized by NAC supplementation, underscoring the antagonistic interplay between redox modulation and cancer cell survival. Furthermore, the timing of NAC administration has emerged as a critical variable influencing therapeutic outcomes. Studies indicate that delayed NAC treatment, rather than concurrent administration with chemotherapy, yields the most favourable results, highlighting the importance of temporal dynamics in NAC’s modulation of redox homeostasis.

Taken together, these findings suggest that while NAC holds therapeutic promise in alleviating treatment-associated toxicities, its application in ovarian cancer requires careful consideration of tumour subtype, redox context, and treatment timing. Future research incorporating subtype-specific models and clinical trials will be essential to delineate the precise role of NAC and optimize its integration into ovarian cancer treatment regimens.

## Figures and Tables

**Table 1 •:** Evidence of the effects of N-Acetyl Cysteine (NAC) in ovarian cancer treatment from preclinical studies.

Study model (cell line/animal)	Effects of NAC	Mechanism	Reference
**In vitro studies**			
A2780 (Cisplatin-sensitive) A2780 cis (Cisplatin-resistant)	NAC has an anti-apoptotic effect when treated in combination with cytotoxic agent MB-591 in A2780 cells. NAC inhibits the autophagic process by MB-591 in A2780 cis cells.	NAC induces XIAP and APAF-1 apoptotic inhibition protein expression in A2780 cells. NAC increased phosphorylation of PTEN while decreased phosphorylation of ULK1 in A2780 cis cells.	[14]
CaOV3	Pre-treatment of NAC prior to Doxorubicin enhances treatment efficacy.	NAC pre-treatment enhances ATM, H2AX and p53 phosphorylation and p53 acetylation involved in apoptosis.	[15]
OVISR, KOC-7	NAC reverses the effects of cytotoxic cysteine deprivation by SAS and PAG combination treatment.	Pretreatment of NAC increased the intracellular cysteine pool and rescued GSH levels.	[16]
SKOV3, TOV21G	NAC treatment reversed the toxic effects of Mirk inhibition and prevented cell loss induced by cisplatin treatment.	NAC decreased ROS levels, reversing the cisplatin-sensitizing effects of Mirk inhibition.	[17]
ES2, OVCAR3, OVCAR8, A2780, A2780 cis	Cysteine allows cells to adapt to the adverse hypoxic environment and protects from carboplatin cytotoxicity	Cysteine acts as a redox buffer, sulphur rapidly binds to carboplatin avoiding DNA damage and evading apoptosis	[18]
**In vivo studies**			
Rats	A 10 h delay of NAC addition after cisplatin treatment prevented apoptosis and dendritic spine loss in hippocampal neurons without affecting cisplatin-induced cytotoxicity in ovarian cancer cells.	NAC acts as a source of L-cysteine for increased GSH biosynthesis in the brain and scavenges oxidants directly through its thiol group.	[5]
Rats	NAC administration inhibited radiotherapy-induced premature ovarian failure and preserved ovarian cells during y-radiation therapy.	NAC alleviated oxidative damage by increased glutathione peroxidase activity and decreased NADPH oxidase subunits expression. NAC also upregulated VEGF expression and suppressed NOX4/MAPK/p53 apoptotic signalling	[19]
Mice	Pretreatment of NAC was protective against x-irradiation through inhibition of oxidative stress.	NAC decreased ROS levels, resulting in less DNA damage. NAC inhibited apoptosis by decreasing levels of cytochrome c and caspase 3.	[20]

**Table 2 •:** Active clinical trials utilizing PD-1 inhibitors in ovarian cancer.

Trial number	Phase	Indications	Disorder	Sponsor (site)	Status	Reference
NCT05200559	1/2	Pembrolizumab	Recurrent/Metastatic Solid Tumors	Alexander B Olawaiye, MD, (University of Pittsburgh)	Recruiting	[50]
NCT03651206	2/3	Dostarlimab + Niraparib	Recurrent Ovarian Carcinosarcoma	ARCAGY/GINECO GROU (France)	Active, not recruiting	[51]
NCT05271318	1/2	Pembrolizumab	Ovarian Cancer	TILT Biotherapeutics Ltd. (USA, Finland)	Recruiting	[52]
NCT05538091	2	Vismodegib + Atezolizumab	Platinum resistant Ovarian, fallopian tube and primary peritoneal cancer	Ronald Buckanovich, (University of Pittsburgh)	Recruiting	[53]
NCT04840589	1	ZEN003694 + Nivolumab, +/− Ipilimumab	Solid Tumours	National Cancer Institute (NCI) (USA)	Recruiting	[54]
NCT04954599	1/2	CP-506 (HAP)	Solid Tumours	Maastricht University Medical Center (Belgium, Netherlands)	Recruiting	[55]
NCT05293496	1	MGC018 + Lorigerlimab	Solid Tumours	MacroGenics (Canada)	Active, not recruiting	[56]
NCT05086692	1/2	MDNA11 + Pembrolizimumab	Advanced Solid Tumours	Medicenna Therapeutics, Inc. (USA)	Recruiting	[57]
NCT04332653	1/2	NT-I7 + Pembrolizumab	Relapsed/Refractory Advanced Solid Tumours	NeoImmuneTec (USA)	Active, not recruiting	[58]
NCT03872947	1	TRK-950 + Nivolumab/Pembrolizumab	Advanced Solid Tumours	Toray Industries, Inc. (USA)	Active, not recruiting	[59]
NCT03522246	3	Rucaparib + Nivolumab	Ovarian Cancer	pharmaand Gmb (238 global locations)	Active, not recruiting	[60]

**Table 3 •:** Active clinical trials utilizing CAR-T cell therapy in ovarian cancer.

Trial number	Phase	Indications	Disorders	Sponsor (site)	Status	Reference
NCT06215950	1	CD70-targeted CAR-T	CD70-positive Advanced/Metastatic Gynecological Cancer	Chongqing Precision Biotech Co., Ltd. (China)	Recruiting	[61]
NCT06010875	1	CD70-targeted CAR-T	CD70-positive Advanced/Metastatic Solid Tumours	Chongqing Precision Biotech Co., Ltd. (China)	Recruiting	[62]
NCT05225363	1	TAG72-chimeric antigen receptor (CAR) T cell	Advanced Epithelial Ovarian Cancer	City of Hope Medical Center (USA)	Recruiting	[63]
NCT05518253	1	CD70-targeted CAR-T	CD70-positive Advanced/Metastatic Solid Tumours	Weijia Fang, MD (China)	Recruiting	[64]
NCT05211557	1/2	Human B7H3 CAR-T cells	Recurrent Malignant Ovarian Cancer	The Affiliated Hospital of Xuzhou Medical University (China)	Recruiting	[65]
NCT05239143	1	P-MUC1C-ALLO1	Advanced Metastatic Solid Tumours	Poseida Therapeutics, Inc. (USA)	Active, not recruiting	[66]
NCT05963100	1/2	TCR-like CAR-T Cell Targeted MSLN	Ovarian Cancer	Zhongda Hospital (China)	Active, not recruiting	[67]
NCT05568680	1	SynKIR-110, Autologous T Cells Transduced With Mesothelin KIR-CAR	Mesothelin-Expressing Advanced Ovarian Cancer, Cholangiocarcinoma, or Mesothelioma	Verismo Therapeutics (USA)	Recruiting	[68]
NCT05274451	1	LYL797, ROR1-Targeting CAR T Cells	Relapsed and/or Refractory Solid-Tumour Malignancies	Lyell Immunopharma, Inc. (USA)	Active, not recruiting	[69]
NCT04511871	1	Autologous CCT303–406 Chimeric Receptor (CAR) T Cells	Relapsed or Refractory HER2 Positive Solid Tumours	Shanghai PerHum Therapeutics Co., Ltd. (China)	Active, not recruiting	[70]
NCT06646627	1	Autologous B7-H3 Chimeric Receptor (CAR) T Cells	Recurrent, Platinum Resistant Ovarian Tumours	Crystal Mackall, MD (USA)	Recruiting	[71]
NCT03907527	1	Autologous PRGN-3005 CAR T Cells	Advanced Stage Platinum Resistant Ovarian Cancer Patients	Precigen, Inc. (USA)	Active, not recruiting	[72]
NCT04670068	1	Activated T-cells Targeting the B7-H3 Antigen	Recurrent Epithelial Ovarian Cancer	UNC Lineberger Comprehensive Cancer Center (USA)	Active, not recruiting	[73]
NCT06469281	1	(27T51) Anti-MUC16 CAR T Cell	Recurrent or Refractory Epithelial Ovarian, Primary Peritoneal, or Fallopian Tube Cancer	Regeneron Pharmaceuticals (USA)	Recruiting	[74]
NCT05057715	1	Human Chimeric Antigen Receptor Modified T Cells (huCART-meso) + VCN-01	Pancreatic and Serous Epithelial Ovarian Cancer	University of Pennsylvania (USA)	Active, not recruiting	[75]

**Table 4 •:** Active clinical trials utilizing adoptive cell therapy in ovarian cancer.

Trial number	Phase	Indications	Disorders	Sponsor (site)	Status	Reference
NCT03412877	2	Autologous T-Cells Genetically Engineered to Express T-Cell Receptors Reactive Against Neoantigens	Metastatic Cancer	National Cancer Institute (NCI) (USA)	Recruiting	[76]
NCT05194735	1/2	Autologous T Cells Engineered Using the Sleeping Beauty System to Express T-Cell Receptors (TCRs) Reactive Against Cancer-specific Mutations	Solid Tumours	Alaunos Therapeutics (USA)	Active, not recruiting	[77]
NCT05397093	1	ITIL-306	Advanced Solid Tumours	Instil Bio (USA)	Active, not recruiting	[78]
